# Eroded plaques involving the breasts: a unique location of pemphigus vulgaris^☆^^☆☆^

**DOI:** 10.1016/j.abd.2020.03.010

**Published:** 2020-07-15

**Authors:** Fernando Garcia-Souto

**Affiliations:** Department of Dermatology, Valme University Hospital, Seville, Spain

Dear Editor,

Pemphigus represents a group of infrequent autoimmune bullous diseases that affect the skin and mucous membranes, characterized by intraepidermal acantholytic blisters. The process of acantholysis is induced by the binding of circulating immunoglobulin G autoantibodies to intercellular adhesion molecules.^1^ Precipitating factors of pemphigus diseases are poorly understood. A unique case of trauma-induced pemphigus vulgaris is reported.

A woman in her 50 s with no relevant past medical history presented with a two-month history of malaise and mildly painful cutaneous lesions predominantly involving both breasts. She denied using topical agents, oral medications, or preceding trauma. However, she did report wearing a tight bra frequently. Physical examination revealed bilateral, strikingly symmetric, well-delimited eroded plaques on both breasts (Fig. 1). In addition, the patient presented a few similar, smaller, isolated eroded plaques distributed on her back. The mucosae were spared. A 4-mm punch skin biopsy was performed.

**Figure 1:**
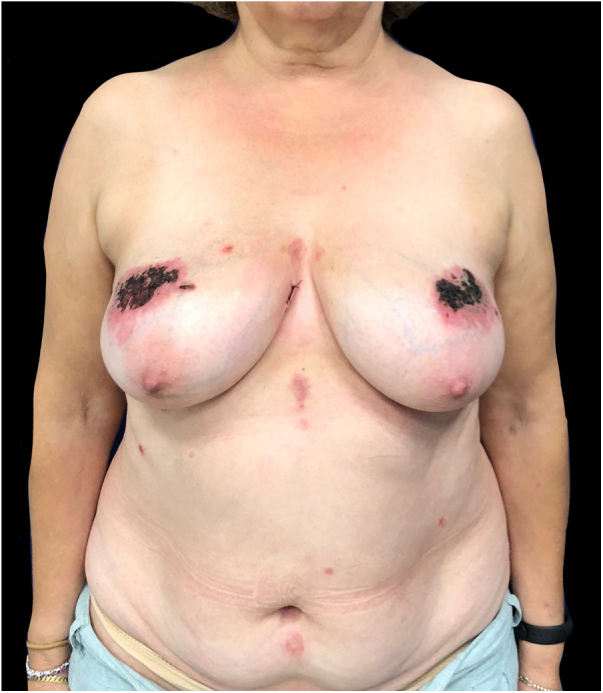
Physical examination revealed bilateral, strikingly symmetric, well-delimited eroded plaques on both breasts.

Histopathology showed an intraepidermal blister with suprabasal acantholysis (Fig. 2A). An unusual dense predominantly lymphocytic infiltrate was also observed within the dermis (Fig. 2B). In addition, direct immunofluorescence microscopy from the perilesional skin showed abnormal intercellular deposits of IgG and C3. Thus, on the basis of clinical, histopathology, and direct immunofluorescence findings, a diagnosis of trauma-induced pemphigus vulgaris was made.

**Figure 2:**
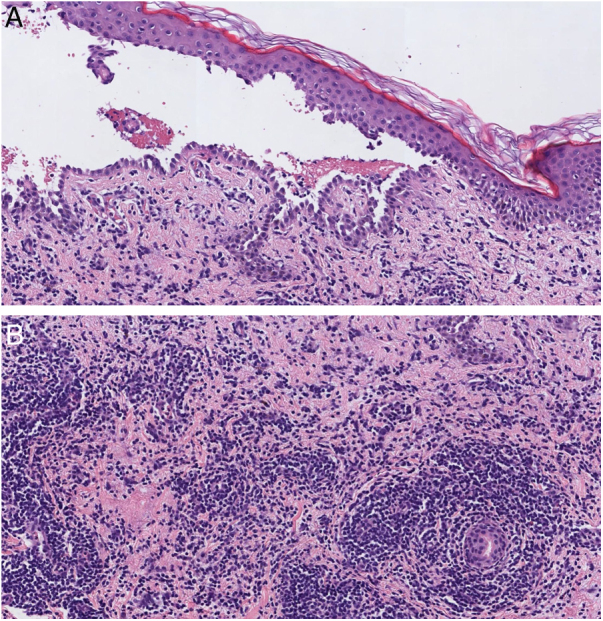
(A), Histopathology showed intraepithelial blister with acantholysis just above the basal keratinocytes and chronic inflammation (Hematoxylin & eosin, ×20). (B), A closer view of the dermis showed an unusual dense inflammatory infiltrate consisting mainly of lymphocytes with some isolated eosinophils (Hematoxylin & eosin, ×20).

Both genetic and environmental factors may influence the development of pemphigus. However, studies on the role of trauma as a triggering factor in pemphigus are limited. The Koebner phenomenon is well known in dermatology. It involves several cutaneous diseases, among them psoriasis, vitiligo, and lichen planus.^2^ Nevertheless, this phenomenon is seldom described in bullous autoimmune disorders such as pemphigus.

Trauma-induced pemphigus has been traditionally reported mainly after a surgery or radiation.^3^ An interesting retrospective study of trauma-induced pemphigus found major surgery as the most frequent trigger.^4^ In addition, authors suggest that more conventional procedures such as periodontal procedures, blunt trauma, or even laser surgery could trigger a pemphigus. The present patient reinforces this latter suggestion, given that the only possible clear trigger might have been the continued friction with the bra.

The lag period between the exposure to trauma and the koebnerization of typical lesions is generally between ten and 20 days.^5^ The pathogenesis of trauma-induced pemphigus is unknown and probably multifactorial, involving genetic factors, immunological factors, and environmental triggers. A possible explanation could be the exposure of pemphigus antigens in traumatized epithelium in genetically predisposed patients.

However, the present patient exhibited the peculiarity of an intense inflammatory infiltrate in the dermis rarely seen previously. According to this case, an atypical intense inflammation could be a distinct histopathological feature of trauma-induced pemphigus. Further research is necessary to ascertain if greater inflammation in dermis is more related to trauma-induced pemphigus as opposed to those not related to trauma.

In conclusion, this report describes a presumably new case of trauma-induced pemphigus. To the author’s knowledge, there are no prior reports of pemphigus involving predominantly the breasts with such a peculiar disposition. It is proposed that a hypothetical continuous trauma with the bra could explain this unique location.

## Financial support

None declared.

## Author's contributions

Fernando Garcia-Souto: Conception and planning of the manuscript; writing and critical revision of the manuscript; approval of the final version.

## Conflicts of interest

None declared.
